# A New Method of Saving Life at Sea—The Fell Submarine Life Preserver

**Published:** 1916-05

**Authors:** George Edward Fell

**Affiliations:** Buffalo, N. Y.


### A New Method of Saving Life at Sea—The Fell Submarine Life Preserver.

GEORGE EDWARD FELL, F. R. M. S., Buffalo, N. Y.

Outside of the life boats, rafts and ordinary cork "life preservers" there is nothing effectual for those suddenly driven overboard, into stormy waters. The cork jackets may keep them above the water, but with a light sea, with white caps, or a brisk wind and driving spray, the best of swimmers, frequently succumb, and there is no chance for the poor women and children. They are doomed from the first.

The difficult problem of providing those who are subjected to such conditions, with a continual supply of fresh air, even when submerged in a heavy sea, has been clearly and practically solved. This apparatus provides an air supply, floating well above the water, and moving vertically with its fluctuating surface. It is well away from the person of the user and independent of his movements, working upon an entirely new method of flotation, being perpendicular in the water, and thus presenting a minimum cross-section of surface to the waves and combers. Every feature of this wonderful apparatus has been the subject of deep thought for many months on the part of its inventor and patentees, one of whom was a United States hydrographical engineer for many years and an adept in everything pertaining to lake and water problems. The simplicity and consequent practicability constitute a most important feature of the apparatus. It can be universally utilized. The name given to this apparatus by its inventors is,

The FELL SUBMARINE LIFE PRESERVER.

It is inexpensive, may be manufactured quickly in large numbers; easily utilized by those who cannot swim; is quickly applied to the person, and through the entirely original design of the air supply Float in a heavy sea way will not lose its protective respiratory features. The three essential features of this apparatus are the,

AIR SUPPLY FLOAT. AIR SUPPLY TUBE.

The air supply float is made of galvanized iron.

The air cupply tube, of a long lived combination rubber tube or metallic tubing almost indestructible, may be used and may be obtained any where in the mercantile world.

### FACE MASK.

The face mask is made of galvanized iron with the rim or face connection of a specially devised plastic material, originated after many experiments by the inventor. This substance has the property of slight agglutinazation when applied to the face, a feature of great value. It is not affected by the water. It does not become dry and brittle as thin shavings exposed to the air for months are yet plastic and apparently have not changed. A rubber air inflated mask that will fit most faces might be used for temporary purposes.

The mask prepared with the plastic material retains the contour of the face for a long time, and the owner of the apparatus may keep it in condition for years. I have used one for months and can attach it any time with the assurance that it will be impermeable to leaking.

This however is a matter of little consequence as provision is made for quickly emptying the mask, air-tube or air supply float of water that by accident or intention might get into them. The mask is quickly and securely applied to the face, by a cap from which ''military belting'' passes around. It is held tightly at the will of the wearer, so that the hands and arms are free to move about, and it is quickly released. If you are desirous of breathing the fresh air for a moment just press the lower edge of the mask out from the chin, or retract the chin, between seas and replace it quickly, without releasing the spring buckle. If the air float becomes completely filled with water, just tip it over with tip of extension within a foot of the water and blow through mask and air tube. The water will quickly flow out of the air float; release the float to upright position and respiration can be at once renewed. This is seldom needed.

### AIR SUPPLY FLOAT.

Three types devised—one for the tourist, yachtsman, and general traveler on steamboats. This outfit weighs about nine pounds, is encased in a canvas sack of small dimensions and can be placed under the berth or hung on the wall.

Another form for use on war vessels, transports, and auxiliary vessels. Its righting weight is within its tube, in which for carrying purposes, is also housed the air supply and extension tubes, which are readily accessible. With a cork jacket applied, the marine or soldier with mask attached, takes the float in one hand and goes overboard with the assurance that he has a chance for his life never before possible in a sea way. A third type, quite light, for carrying, has a water-weight, and will be useful at beaches and summer water resorts.

### AIR SUPPLY TUBE.

This connects float and face mask. It is of a size to supply sufficient air for respiratory purposes, and of a length to permit the pure inspired air to overcome the vitiating of the air in the mask, air-tube, and air float, and it does this effectually.

### BUOYANCY OF AIR SUPPLY FLOAT.

Constructed so that when the whole apparatus is filled with water from tip of extension tube away above the water, the air chamber, to air tube and face mask, it will not sink. In other words, even through carelessness, you cannot lose the outfit.

The detail of this valuable apparatus, would require more space than I can give here. Those who obtain it can readily grasp the simple requirements of practical usage, and acquire a confidence that will enable them to be quite comfortable in a sea way. If exhausted, they can sleep for hours, if the temperature of the water is not too low, and have the sea washing over them at frequent intervals. It is a great step in advance with possibilities most interesting and valuable. For instance:—

Food and water supply may be made positive through the upright, tubular principle, which permits a wide range of function. The gurgle call of the sea may be heard for a long distance. A white flag on the extension by day, red fire by night, a shark pike for marauders of the sea and other robbers, and many other novel ideas that have arisen.

To save human lives is the purpose of the invention primarily, and the hope of the inventor is that it may equal the record of that other boon to humanity, "Mechanical Respiration," which he gave to the world and has resulted in the saving of more than fifty thousand human lives.

209 Porter Avenue, Buffalo, N. Y.

---

**Anti-Typhoid Vaccination.** C. C. Burlingame, Fergus Falls, Minn., St. Paul Med. Jour., Jan. 1916, reports the results of 2200 vaccinations in the Fergus Falls State Hospital for the Insane. The vaccinations have been done in groups beginning in 1912. Since the fall of 1913, about three quarters of the inmates have been vaccinated. 22 cases of typhoid have occurred in all, and all among the unvaccinated, though the living conditions were the same.

